# The Burden of Unintentional Falls Among Adolescents in Iran During 1990–2021 Based on the Global Burden of Disease Study

**DOI:** 10.1155/ijpe/9693349

**Published:** 2026-07-21

**Authors:** Habibollah Azarbakhsh, Andishe Hamedi, Sanaz Amiri, Seyed Parsa Dehghani, Mohadeseh Ghanbari-Jahromi, Fatemeh Rezaei

**Affiliations:** ^1^ Department of Community Medicine, School of Medicine, Ahvaz Jundishapur University of Medical Sciences, Ahvaz, Iran, ajums.ac.ir; ^2^ Department of Epidemiology, Student Research Committee, Shiraz University of Medical Sciences, Shiraz, Iran, sums.ac.ir; ^3^ School of Medicine, Shiraz University of Medical Sciences, Shiraz, Iran, sums.ac.ir; ^4^ Department of Epidemiology, Research Center for Social Determinants of Health, Jahrom University of Medical Sciences, Jahrom, Iran, jums.ac.ir; ^5^ Department of Community Medicine, Research Center for Social Determinants of Health, Jahrom University of Medical Sciences, Jahrom, Iran, jums.ac.ir

**Keywords:** disability-adjusted life years, Global Burden of Diseases, Iran, morbidity, mortality, unintentional fall

## Abstract

**Introduction:**

Children, due to their naturally active behavior, and adolescents, because of their exploratory tendencies and engagement in risky behaviors, are more vulnerable to injuries such as falls. The purpose of this study is to investigate the trend of morbidity, mortality, and burden of unintentional fall in Iran.

**Methods:**

Indicators including prevalence, incidence, death, years of life lost (YLL), years lived with disability (YLD), and DALY for two genders in Iran were extracted from GBD 2021. A joinpoint regression analysis was used to examine the pattern of changes.

**Results:**

During the study period (1990–2021), 6583 deaths from unintentional fall occurred in the adolescents in Iran. Of these, 77.1% (*n* = 5077) cases occurred in boys. The incidence trend has been decreasing in boys and increasing in girls. The prevalence trend in boys and girls has been decreasing. The mortality trend in boys and girls has been decreasing. The trend in the DALY rate in boys and girls has been decreasing. According to the joinpoint regression analysis, the trend in the DALY rate in boys and girls has been decreasing. AAPC was −1.8 (95% CI: −1.82 to −1.78) for boys and −1.5 (95% CI: −1.52 to −1.48) for girls.

**Conclusions:**

Although incidence, prevalence, mortality, DALY, YLL, and YLD rates have declined over the past three decades, the burden of unintentional falls among adolescents—especially boys—remains substantial. Prioritizing targeted prevention for high‐risk groups and strengthening safety education and environmental safety measures are essential policy actions.

## 1. Introduction

Approximately 26% of the global population consists of children and adolescents [1]. Children, due to their naturally active behavior, and adolescents, because of their exploratory tendencies and engagement in risky behaviors, are more vulnerable to injuries such as falls [2]. Unintentional injuries resulting from falls are among the leading causes of morbidity and mortality in both children and adults [3]. A fall is defined as “an unintentional descent to the ground, with or without injury” [4]. Falls represent the second leading cause of mortality globally [5] and are the most common reason for emergency department admissions in childhood, as well as the fourth leading cause of trauma overall [6]. Each year, approximately 37.3 million severe falls occur worldwide, resulting in the loss of an estimated 17 million disability‐adjusted life years (DALYs) [5]. Globally, falls rank as the 13th most common cause of DALY loss, with over 50% of fall‐related DALYs occurring in children and adolescents [7]. The incidence of falls in the 10–19 age group increased from 3.35 in 2005 to 3.77 in 2018 [8]. Mortality risk in the 10–14 age group for males and the 15–19 age group for females has decreased to its lowest point but has begun to rise gradually [9]. Various factors contribute to unintentional falls during childhood, including age, sex, geographic region, child development, parental literacy, overcrowded households, unsafe architectural features (e.g., unguarded stairs, windows, and rooftops), unsafe storage of hazardous materials (e.g., kerosene and medications), poorly designed kitchens with access to stoves and knives, and inadequate home lighting [5, 10–13]. Falls among children and adolescents can result in traumatic brain injuries, spinal cord injuries, and damage to internal abdominal organs, and complex skeletal fractures [14].

Several studies have identified children and adolescents as high‐risk populations for falls. Research on injuries in low‐ and middle‐income countries revealed that 56% of injuries in children were fall‐related [15]. A study from India found that falls were the most common type of domestic injury among children aged 0–14 years [16]. Another study in 2021 indicated that 26.3% of all unintentional injuries in adolescents were due to falls [17]. Similarly, a study in India reported that fall‐related injuries had the highest prevalence (3.38%), followed by road traffic injuries (RTIs) at 1.62% [18]. Dávila‐Cervantes et al. [19] reported that interpersonal violence, RTIs, falls, and self‐harm accounted for 8 out of 10 injury‐related deaths in 2019, with the injury burden being higher in males across all age groups. According to the World Health Organization (WHO), motor vehicle accidents, falls, and interpersonal violence are among the top 20 causes of death and disability across all age groups globally [14].

Falls are one of the most common causes of unintentional injury among adolescents and significantly contribute to morbidity, mortality, and both physical and psychological disabilities in this age group. Adolescents aged 10–19 are particularly at risk due to behavioral changes and increased engagement in high‐risk activities. Analyzing data from the Global Burden of Disease (GBD) Study allows for a comprehensive understanding of the distribution of fall‐related indicators—such as morbidity, mortality, and DALYs—across both sexes, various age groups within the 10–19 range, and different geographical regions. Therefore, a detailed analysis of GBD data in this context is crucial for developing effective preventive interventions and public health policies targeting adolescent health.

## 2. Methods

The present study is a secondary data analysis based on the ecological longitudinal data and modeled estimates provided by the GBD 2021, which were conducted to investigate the epidemiological indicators of unintentional falls in children aged 10–9 years in Iran from 1990 to 2021.

The GBD 2021 report covers 371 diseases and injuries in 204 countries and territories, including estimates from different models for disease and injury outcomes. These estimates are used for prevalence, incidence, mortality, years of life lost (YLLs), years of life with disability (YLDs), and DALYs. Such estimates are disaggregated by cause, age, sex, year, and location [20]. YLLs are the number of years lost due to premature death associated with a disease, calculated by multiplying the number of deaths by the standard life expectancy at the age at which death occurred. YLDs are the number of YLL in good health due to disability or due to disease, calculated by multiplying the prevalence of the disease by the relevant disability weights. DALYs measure the total years of healthy life lost from disease onset to death. DALYs were calculated as the sum of YLDs and YLLs. The 95% uncertainty intervals (UI) for all final estimates are reported as the 2.5th and 97.5th percentile values [21].

GBD 2021 also uses the International Classification of Diseases 10 (ICD‐10), and falls are identified by codes (W00‐W19) in this data. For this study, we extracted incidence, mortality, and DALYs by age and sex from 1990 to 2021.

### 2.1. Statistical Analysis

Joinpoint regression analysis was also used to analyze trends in epidemiologic indicators of falls. Joinpoint regression is a trend analysis software developed by the U.S. National Cancer Institute for the analysis of SEER data [22].

To ensure model parsimony and mitigate the risk of overfitting, the search for optimal joinpoints was constrained to a maximum of five, and the final number of segments was determined using a permutation test. A log‐linear transformation was applied to the data to account for the multiplicative nature of the trends and to facilitate the estimation of the annual percent change (APC).

For each identified segment, the APC was calculated, whereas the average annual percent change (AAPC) was computed as a weighted average of the APCs across the entire study period, with weights proportional to the length of each joinpoint segment. The APC served to test the null hypothesis of a zero‐percentage change; positive and negative values were interpreted as increasing and decreasing trends, respectively. In instances where the model identified no breakpoints, indicating no significant shift in the trend, the APC remained constant throughout the study period, rendering it equivalent to the AAPC [23]. We used joinpoint regression software Version 4.9.1.0 to analyze the data in this study.

## 3. Results

### 3.1. Main Causes of Death by Sex and Cause

During the study period (1990–2021), 6583 deaths from unintentional fall occurred in the adolescents in Iran. Of these, 77.1% (*n* = 5077) cases occurred in boys.

### 3.2. Morbidity Trend

**Figure 1:**
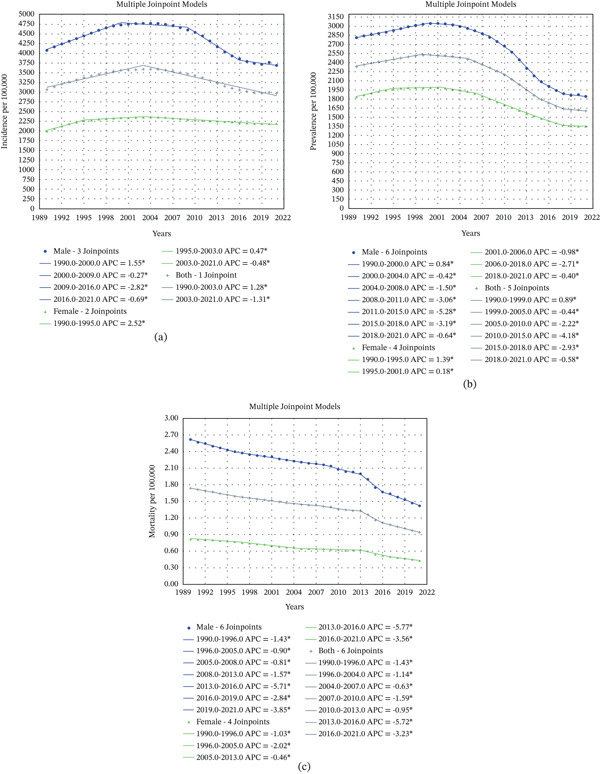
The multiple joinpoint regression model of fall indices. (a) The trend of incidence rate of fall for boys, girls, and both sexes; (b) the trend of prevalence rate of fall for boys, girls, and both sexes; and (c) the trend of mortality rate of fall for boys, girls, and both sexes.

Descriptive statistics of incidence, prevalence, and mortality rates of unintentional fall among adolescents in Iran during the Years 1990, 1995, 2000, 2005, 2010, 2015, and 2021 are shown in Table 1. The results of the joinpoint regression analysis for incidence, prevalence, and mortality rate are shown in Table 2. For boys, three joinpoints and four time periods have been observed. An increasing trend in incidence has been observed during the periods 1990–2000 (first period), and three decreasing trends has been observed during the periods 2000–2009, 2009–2016, and 2016–2021. For girls, two joinpoints and three time periods have been observed. Two increasing trend in incidence has been observed during the periods 1990–1995 (first period) and 1995–2003 (second period), and one decreasing trends has been observed during the period 2003–2021. Based on the results of joinpoint regression, the incidence trend has been decreasing in men and increasing in women. The AAPC was −0.3 (95% CI: −0.35 to −0.25) for boys, 0.2 (95% CI: 0.16–0.24) for girls, and −0.2 (95% CI: −0.25 to −0.15) for both sex (Table 2 and Figure 1).

**Table 1:** The incidence, mortality, and prevalence rate per 100,000 population (95% uncertainty intervals) fall among adolescents in Iran for the selected years from the Global Burden of Disease database.

	Sex	1990	1995	2000	2005	2010	2015	2021
Incidence	**Male**	4082.76 (2901.77–5366.69)	4451.72 (3193.32–5865.04)	4733.26 (3364.34–6250.42)	4767.67 (3369.45–6272.59)	4544.05 (3241.23–5976.30)	3932.38 (2908.27–5091.48)	3692.76 (2783.44–4783.83)
	**Female**	2000.02 (1316.93–2807.43)	2261.32 (1491.64–3161.69)	2339.26 (1557.24–3267.97)	2360.27 (1560.16–3294.17)	2277.34 (1484.43–3189.60)	2233.34 (1500.23–3052.36)	2196.71 (1482.49–3025.83)
	**Both**	3062.90 (2119.25–4062.01)	3368.19 (2369.90–4442.34)	3556.65 (2511.12–4753.10)	3586.14 (2519.98–4753.73)	3429.52 (2396.26–4562.67)	3100.82 (2234.16–4060.55)	2963.60 (2158.48–3893.65)

Prevalence	**Male**	2815.63 (2164.63–3921.67)	2932.50 (2253.31–4084.58)	3047.67 (2325.21–4225.20)	2967.95 (2249.71–4071.65)	2676.40 (2028.68–3661.64)	2088.11 (1598.68–2805.95)	1843.87 (1417.94–2464.45)
	**Female**	1837.81 (1372.62–2676.72)	1966.99 (1468.02–2898.78)	1993.13 (1476.50–2916.75)	1924.09 (1432.27–2771.70)	1707.86 (1279.19–2435.69)	1481.65 (1108.64–2101.23)	1354.96 (1012.59–1934.41)
	**Both**	2336.82 (1776.19–3297.06)	2454.89 (1859.92–(3505.10)	2529.38 (1918.14–3568.99)	2455.63 (1859.14–3425.31)	2200.18 (1651.16–3060.55)	1791.29 (1347.36–2468.87)	1605.58 (1218.75–2208.93)

Mortality	**Male**	2.62 (1.97–3.26)	2.43 (1.84–2.94)	2.32 (1.77–2.86)	2.20 (1.70–2.82)	2.08 (1.61–2.62)	1.74 (1.48–2.21)	1.41 (1.08–1.82)
	**Female**	0.82 (0.61–1.05)	0.78 (0.56–1.03)	0.71 (0.51–0.98)	0.64 (0.46–0.90)	0.61 (0.43–0.83)	0.54 (0.40–0.72)	0.43 (0.33–0.56)
	**Both**	1.74 (1.34–2.07)	1.61 (1.26–1.92)	1.53 (1.20–1.89)	1.43 (1.13–1.82)	1.36 (1.07–1.70)	1.15 (1.00–1.42)	0.93 (0.76–1.16)

**Table 2:** The joinpoint regression model output for the incidence, prevalence, and mortality rate trend analysis of fall among adolescents in Iran between 1990 and 2021 for male, female, and both together based on the Global Burden of Disease database.

	Segments	Male		Female		Both	
		Time interval	APC (95% CI)	Time interval	APC (95% CI)	Time interval	APC (95% CI)
Incidence	**Trend 1**	1990–2000	1.6 ^∗^ (1.40–1.70)	1990–1995	2.5 ^∗^ (2.20–2.80)	1990–2003	1.3 ^∗^ (1.00–1.60)
	**Trend 2**	2000–2009	−0.3 ^∗^ (−0.50, −0.10)	1995–2003	0.5 ^∗^ (0.30–0.70)	2003–2021	−1.3 ^∗^ (−1.50, −1.20)
	**Trend 3**	2009–2016	−2.8 ^∗^ (−3.10, −2.60)	2003–2021	−0.5 ^∗^ (−0.52, −0.48)	—	—
	**Trend 4**	2016–2021	−0.7 ^∗^ (−1.1, −0.5)	—	—	—	—
	**AAPC**	1990–2021	−0.3 ^∗^ (−0.35, −0.25)	1990–2021	0.2 ^∗^ (0.16–0.24)	1990–2021	−0.2 ^∗^ (−0.24, −0.16)

Prevalence	**Trend 1**	1990–2000	0.8 ^∗^ (0.75–0.85)	1990–1995	1.4 ^∗^ (1.20–1.60)	1990–1999	0.9 ^∗^ (0.80–1.00)
	**Trend 2**	2000–2004	−0.4 ^∗^ (−0.60, −0.10)	1995–2001	0.2 ^∗^ (0.10–0.30)	1999–2005	−0.4 ^∗^ (−0.60, −0.20)
	**Trend 3**	2004–2008	−1.5 ^∗^ (−1.80, −1.20)	2001–2006	−1.0 ^∗^ (−1.22, −0.82)	2005–2010	−2.2 ^∗^ (−2.40, −2.00)
	**Trend 4**	2008–2011	−3.1 ^∗^ (−3.40, −2.70)	2006–2018	−2.7 ^∗^ (−2.80, −2.60)	2010–2015	−4.2 ^∗^ (−4.50, −4.00)
	**Trend 5**	2011–2015	−5.3 ^∗^ (−5.60, −5.10)	2018–2021	−0.4 ^∗^ (−0.60, −0.20)	2015–2018	−2.9 ^∗^ (−3.60, −2.20)
	**Trend 6**	2015–2018	−3.2 ^∗^ (−3.50, −2.90)	—	—	2018–2021	−0.6 ^∗^ (−1.00, −0.10)
	**Trend 7**	2018–2021	−0.6 ^∗^ (−1.00, −0.35)	—	—	—	—
	**AAPC**	1990–2021	−1.3 ^∗^ (−1.35, −1.25)	1990–2021	−1.0 ^∗^ (−1.02, −0.98)	1990–2021	−1.2 ^∗^ (−1.22, −1.18)

Mortality	**Trend 1**	1990–1996	−1.4 ^∗^ (−2.00, −0.80)	1990–1996	−1.0 ^∗^ (−1.40, −0.00)	1990–1996	−1.4 ^∗^ (−2.10, −0.80)
	**Trend 2**	1996–2005	−0.9 ^∗^ (−1.80, −0.50)	1996–2005	−2.0 ^∗^ (−2.90, −1.80)	1996–2004	−1.1 ^∗^ (−1.80, −0.50)
	**Trend 3**	2005–2008	−0.8 ^∗^ (−1.40, −0.50)	2005–2013	−0.5 ^∗^ (−0.80, −0.00)	2004–2007	−0.6 ^∗^ (−1.80, −0.30)
	**Trend 4**	2008–2013	−1.6 ^∗^ (−1.90, −0.50)	2013–2016	−5.8 ^∗^ (−6.40, −4.30)	2007–2010	−1.6 ^∗^ (−2.00, −0.40)
	**Trend 5**	2013–2016	−5.7 ^∗^ (−6.10, −1.50)	2016–2021	−3.6 ^∗^ (−4.10, −2.40)	2010–2013	−0.9 ^∗^ (−6.00, −0.50)
	**Trend 6**	2016–2019	−2.8 ^∗^ (−5.70, −2.40)	—	—	2013–2016	−5.7 ^∗^ (−6.10, −2.90)
	**Trend 7**	2019–2021	−3.8 ^∗^ (−4.60, −3.10)	—	—	2016–2021	−3.2 ^∗^ (−4.00, −2.60)
	**AAPC**	1990–2021	−2.0 ^∗^ (−2.02, −1.98)	1990–2021	−2.0 ^∗^ (−2.04, −1.96)	1990–2021	−2.0 ^∗^ (−2.03, −1.97)

*Note:* Asterisk “ ^∗^” denotes significant at 0.05 level.

Abbreviation: APC, annual percentage change.

For prevalence, in boys, six joinpoints and seven time periods have been observed. An increasing trend in prevalence has been observed during the periods 1990–2000 (first period), and six decreasing trends has been observed during the periods 2000–2004, 2004–2008, 2008–2011, 2011–2015, 2015–2018, and 2018–2021. For girls, four joinpoints and five time periods have been observed. Two increasing trend in prevalence has been observed during the periods 1990–1995 (first period) and 1995–2001 (second period), and three decreasing trends has been observed during the periods 2001–2006, 2006–2018, and 2018–2021. Based on the results of joinpoint regression, the prevalence trend in boys, girls, and both sexes has been decreasing. The AAPC was −1.3 (95% CI: −1.35 to −1.25) for boys, −1.0 (95% CI: −1.02 to −0.98) for girls, and −1.2 (95% CI: −1.22 to −1.18) for both sex (Table 2 and Figure 1).

### 3.3. Mortality Trend

For mortality, in boys, six join points and seven time periods have been observed. Seven decreasing trends have been observed during the periods 1990–1996, 1996–2005, 2005–2008, 2008–2013, 2013–2016, 2016–2019, and 2019–2021. For girls, four join points and five time periods have been observed. Five decreasing trends have been observed during the periods 1990–1996, 1996–2005, 2005–2013, 2013–2016, and 2016–2021. Based on the results of joinpoint regression, the mortality trend in boys, girls, and both sexes has been decreasing. The AAPC was −2.0 (95% CI: −2.02 to −1.98) for boys, −2.0 (95% CI: −2.04 to −1.96) for girls, and −2.0 (95% CI: −2.03 to −1.97) for both sexes (Table 2 and Figure 1).

### 3.4. DALY, YLL, and YLD Trends

**Figure 2:**
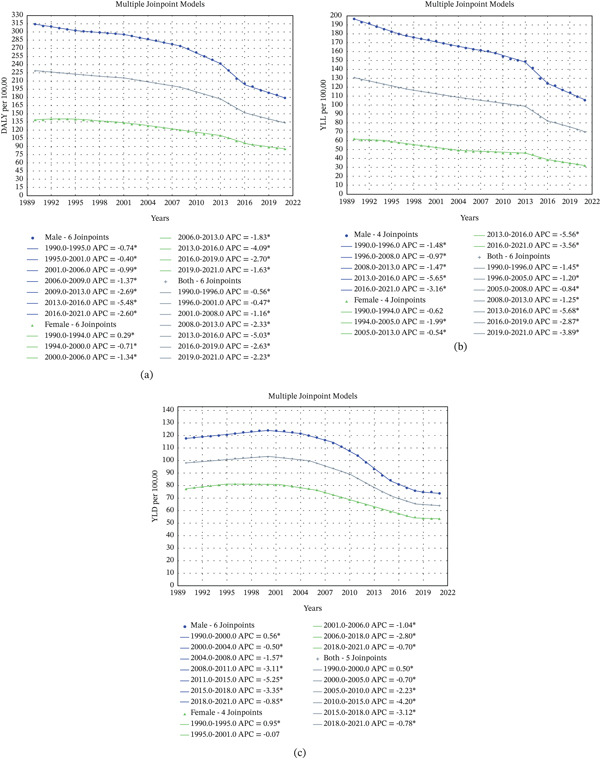
The multiple joinpoint regression model of the fall indices. (a) The trend of DALY rate of fall for boys, girls, and both sexes; (b) the trend of YLL rate of fall for boys, girls, and both sexes; and (c) the trend of YLD rate of fall for boys, girls, and both sexes.

Descriptive statistics of DALY, YLL, and YLD rates of unintentional fall among adolescents in Iran during the Years 1990, 1995, 2000, 2005, 2010, 2015, and 2021 are shown in Table 3. The number of DALY has decreased from 30,616.09 (95% UIs: 24,920.35–37,404.45) in 1990 to 16,439.77 (95% UI: 13,377.56–20,426.61) in 2021. The total number of DALY during the study period was 914,928 years. Of these, 491,832 years (53.8%) were related to YLL and 423,096 related to YLD. Moreover, 636,476 (69.6%) of those years were in boys. According to joinpoint regression analysis, the trend in the DALY rate in boys, girls, and both sexes has been decreasing. AAPC was −1.8 (95% CI: −1.82 to −1.78) for boys, −1.5 (95% CI: −1.60 to −1.40) for girls, and −1.7 (95% CI: −1.72 to −1.68), for both sexes (Table 4 and Figure 2).

**Table 3:** The years of life lost (YLL), years lived with disability (YLD), and disability‐adjusted life years (DALY) rates per 100,000 population (95% uncertainty intervals [UI]) of fall among adolescents in Iran for the selected years from the Global Burden of Disease database.

	Sex	1990	1995	2000	2005	2010	2015	2021
YLL	**Male**	196.73 (147.98–244.60)	182.39 (138.50–220.90)	173.15 (132.75–213.69)	163.91 (126.38–209.32)	154.62 (119.46–194.29)	129.81 (110.05–165.25)	105.45 (81.01–136.23)
	**Female**	61.99 (46.77–79.58)	59.01 (42.42–78.11)	53.85 (38.69–74.06)	48.15 (34.92–67.37)	46.25 (32.74–62.09)	40.81 (30.76–54.72)	32.30 (25.44–42.33)
	**Both**	130.75 (100.78–155.73)	121.35 (94.63–144.76)	114.52 (90.04–142.00)	107.09 (84.59–135.55)	101.34 (80.01–126.81)	86.25 (75.02–106.24)	69.80 (56.85–86.53)

YLD	**Male**	117.70 (79.42–168.43)	120.60 (80.40–173.61)	123.90 (82.84–178.95)	120.02 (81.12–174.46)	107.89 (72.66–156.02)	84.24 (56.94–121.51)	73.63 (48.62–106.54)
	**Female**	77.25 (50.50–113.00)	80.89 (52.86–118.44)	80.90 (52.34–119.39)	77.64 (49.91–114.01)	68.66 (43.94–100.84)	59.37 (38.24–87.23)	53.62 (34.31–78.89)
	**Both**	97.89 (65.16–141.40)	100.96 (66.70–147.41)	102.77 (68.21–148.52)	99.22 (66.18–145.02)	88.60 (58.99–129.23)	72.07 (48.20–105.21)	63.88 (42.05–93.20)

DALY	**Male**	314.43 (253.23–383.59)	302.99 (245.04–371.33)	297.06 (237.68–368.40)	283.93 (230.90–356.21)	262.52 (213.66–329.15)	214.06 (177.61–262.54)	179.08 (144.61–217.17)
	**Female**	139.24 (109.74–178.56)	139.90 (108.06–181.61)	134.76 (103.15–177.84)	125.79 (95.88–166.65)	114.92 (87.13–150.67)	100.18 (76.89–132.10)	85.93 (64.87–113.94)
	**Both**	228.65 (186.11–279.35)	222.31 (179.99–274.12)	217.29 (175.13–271.87)	206.31 (165.73–258.71)	189.94 (154.32–236.98)	158.32 (131.35–196.38)	133.68 (108.78–166.10)

**Table 4:** The joinpoint regression model output for the trend analysis of the years of life lost (YLL), years lived with disability (YLD), and disability‐adjusted life years (DALY) of fall among adolescents in Iran between 1990 and 2021 for male, female, and both based on the Global Burden of Disease database.

Index	Segments	Male		Female		Both	
		Time interval	APC (95% CI)	Time interval	APC (95% CI)	Time interval	APC (95% CI)
YLL	**Trend 1**	1990–1996	−1.5 ^∗^ (−2.30, −1.20)	1990–1994	−0.6 (−1.30, −0.60)	1990–1996	−1.5 ^∗^ (−2.20, −0.70)
	**Trend 2**	1996–2008	−1.0 ^∗^ (−1.10, −0.40)	1994–2005	−2.0 ^∗^ (−2.40, −1.80)	1996–2005	−1.2 ^∗^ (−1.90, −0.70)
	**Trend 3**	2008–2013	−1.5 ^∗^ (−2.00, −1.20)	2005–2013	−0.5 ^∗^ (−0.80, −0.20)	2005–2008	−0.8 ^∗^ (−1.70, −0.50)
	**Trend 4**	2013–2016	−5.7 ^∗^ (−6.00, −4.80)	2013–2016	−5.6 ^∗^ (−6.10, −4.40)	2008–2013	−1.3 ^∗^ (−1.70, −0.50)
	**Trend 5**	2016–2021	−3.2 ^∗^ (−3.50, −2.60)	2016–2021	−3.6 ^∗^ (−4.00, −2.60)	2013–2016	−5.7 ^∗^ (−6.10, −1.10)
	**Trend 6**	—	—	—	—	2016–2019	−2.9 ^∗^ (−5.80, −2.40)
	**Trend 7**	—	—	—	—	2019–2021	−3.9 ^∗^ (−4.70, −3.10)
	**AAPC**	1990–2021	−2.0 ^∗^(−2.02, −1.98)	1990–2021	−2.1 (−2.12, −2.08)	1990–2021	−2.0 (−2.10, −1.90)

YLD	**Trend 1**	1990–2000	0.6 ^∗^ (0.50–0.60)	1990–1995	1.0 ^∗^ (0.80–1.10)	1990–2000	0.5 ^∗^ (0.40–0.60)
	**Trend 2**	2000–2004	−0.5 ^∗^ (−0.80, −0.10)	1995–2001	−0.1 (−0.14–0.96)	2000–2005	−0.7 ^∗^ (−0.90, −0.50)
	**Trend 3**	2004–2008	−1.6 ^∗^ (−1.80, −1.30)	2001–2006	−1.0 ^∗^ (−1.20, 0.90)	2005–2010	−2.2 ^∗^ (−2.40, −2.00)
	**Trend 4**	2008–2011	−3.1 ^∗^ (−3.60, −2.70)	2006–2018	−2.8 ^∗^ (−2.90, −2.70)	2010–2015	−4.2 ^∗^ (−4.40, −4.10)
	**Trend 5**	2011–2015	−5.2 ^∗^ (−5.60, −5.10)	2018–2021	−0.7 ^∗^ (−0.90, −0.50)	2015–2018	−3.1 ^∗^ (−3.50, −2.80)
	**Trend 6**	2015–2018	−3.3 ^∗^ (−3.70, −3.00)	—	—	2018–2021	−0.8 ^∗^ (−1.10, −0.40)
	**Trend 7**	2018–2021	−0.9 ^∗^ (−1.20, −0.40)	—	—	—	—
	**AAPC**	1990–2021	−1.5 ^∗^ (−1.52, −1.48)	1990–2021	−1.2 ^∗^ (−1.22, −1.18)	1990–2021	−1.4 ^∗^ (−1.42, −1.38)

DALY	**Trend 1**	1990–1995	−0.7 ^∗^ (−1.20, −0.30)	1990–1994	0.3 ^∗^ (0.00, 0.70)	1990–1996	−0.6 ^∗^ (−0.90, −0.10)
	**Trend 2**	1995–2001	−0.4 ^∗^ (−0.80, −0.00)	1994–2000	−0.7 ^∗^ (−0.90, −0.40)	1996–2001	−0.5 ^∗^ (−1.40, −0.20)
	**Trend 3**	2001–2006	−1.0 ^∗^ (−1.30, −0.30)	2000–2006	−1.3 ^∗^ (−1.60, −0.80)	2001–2008	−1.2 ^∗^ (−1.60, −0.30)
	**Trend 4**	2006–2009	−1.4 ^∗^ (−2.80, −1.00)	2006–2013	−1.8 ^∗^ (−2.00, −1.30)	2008–2013	−2.3 ^∗^ (−2.50, −1.00)
	**Trend 5**	2009–2013	−2.7 ^∗^ (−5.60, −2.50)	2013–2016	−4.1 ^∗^ (−4.30, −1.80)	2013–2016	−5.0 ^∗^ (−5.20, −2.20)
	**Trend 6**	2013–2016	−5.5 ^∗^ (−5.70, −2.50)	2016–2019	−2.7 ^∗^ (−4.20, −2.50)	2016–2019	−2.6 ^∗^ (−5.20, −2.50)
	**Trend 7**	2016–2021	−2.6 ^∗^ (−3.10,−2.20)	2019–2021	−1.6 ^∗^ (−2.30, −1.20)	2019–2021	−2.2 ^∗^ (−2.60, −1.80)
	**AAPC**	1990–2021	−1.8 ^∗^ (−1.82, −1.78)	1990–2021	−1.5 ^∗^ (−1.60, −1.40)	1990–2021	−1.7 ^∗^ (−1.72, −1.68)

*Note:* Asterisk “ ^∗^” denotes significant at 0.05 level.

Abbreviation: AAPC, average annual percentage change.

For boys, six joinpoints and seven time periods have been observed. Seven decreasing trends has been observed during the periods 1990–1995, 1995–2001, 2001–2006, 2006–2009, 2009–2013, 2013–2016, and 2016–2021. For girls, six joinpoints and seven time periods have been observed. An increasing trend in DALY has been observed during the periods 1990–1994 (first period), and six decreasing trends has been observed during the periods 1994–2000, 2000–2006, 2006–2013, 2013–2016, 2016–2019, and 2019–2021.

The number of YLL has decreased from 17,507.90 (95% UIs: 13,495.22–20,852.14) in 1990 to 8583.87 (95% UI: 6991.87–10,641.74) in 2021. The total YLL during the study period was 491,832 years. Of this number, 378,558 (77.0%) years were in boys.

According to joinpoint regression analysis, the trend in the YLL rate in boys, girls, and both sexes has been decreasing. AAPC was −2.0 (95% CI: −2.02 to −1.98) for boys, −2.1 (95% CI: −2.12 to −2.08) for girls, and −2.0 (95% CI: −2.10 to −1.90) for both sexes (Table 4 and Figure 2).

The number of YLD has decreased from 13,108.16 (95% UIs: 8725.39–18,933.67) in 1990 to 7855.89 (95% UI: 5172.09–11,461.84) in 2021. The total YLD during the study period was 423,096 years. Of this number, 257,917 (61.0%) years were in boys.

According to joinpoint regression analysis, the trend in the YLD rate in boys, girls, and both sexes has been decreasing. AAPC was −1.5 (95% CI: −1.52 to −1.48) for boys, −1.2 (95% CI: −1.22 to −1.18) for girls, and −1.4 (95% CI: −1.42 to −1.38) for both sexes (Table 4 and Figure 2).

## 4. Discussion

The findings of this study indicate that unintentional falls remain a major cause of death and disability among adolescents in Iran. Despite significant advances in public health and education, hundreds of adolescents continue to die or become disabled from falls each year over the 32 years studied. Epidemiological trends based on GBD data and joinpoint regression analysis provide a detailed picture of temporal changes in incidence, prevalence, mortality, and disease burden indicators that can guide preventive policies and future interventions.

In this study, 6583 deaths from unintentional falls occurred in the 10‐ to 19‐year‐old age group between 1990 and 2021, with more than three‐quarters (77.1%) of them occurring in boys. This gender difference is consistent with global findings that boys are at higher risk of falling due to more risky behaviors and more physical activity [24]. In addition to behavioral explanations, several sociocultural and environmental factors in Iran may further clarify the considerably higher burden of fall‐related injuries observed in boys. Adolescent boys are more frequently involved in physically demanding tasks, outdoor activities, and informal labor, all of which increase exposure to fall hazards. Moreover, cultural norms in Iranian communities often encourage boys to engage in high‐risk physical activities and provide them with greater autonomy in outdoor environments compared with girls. Environmental and occupational safety gaps—including inadequate use of protective equipment, limited safety oversight in informal work settings, and insufficient safety standards in some urban and rural areas—also disproportionately affect boys. Furthermore, differences in risk perception and lower adherence to safety recommendations among boys, as reported in Iranian studies, may contribute to the observed sex‐specific disparities in mortality and DALYs due to falls [25, 26]. A population‐based study showed that the mortality rate from falls is four times higher in men than in women [27]. In Iran, a study conducted by Azarbakhsh et al. [28] between 2004 and 2019 emphasized that most deaths from unintentional injuries (including falls) occur in boys and the 15‐ to 19‐year‐old age group. Globally, “The global burden of falls: global, regional and national estimates of morbidity and mortality from the Global Burden of Disease Study 2017” found that although the absolute number of deaths and DALYs from falls increased from 1990 to 2017, the age‐standardized rates of mortality and DALYs have decreased slightly. In Brazil, there were 72,234 deaths from falls in all age groups over 16 years between 2008 and 2016. The highest number of deaths was in men aged 15–29 years and in women aged over 80 years. However, there was a decreasing trend in the crude and standardized fall mortality rate in men and an increasing trend in women [29].

In addition, in the present study, the prevalence of falls showed a decreasing trend in both genders, and a significant annual decrease was observed in both groups. This decrease could be due to improved environmental standards, safety training in schools, and preventive interventions. Results of a WHO study on the prevalence, risk factors, and disability associated with fall‐related injuries in older adults in low‐ and middle‐income countries found that the prevalence of fall‐related injuries ranged from 6.6% in India to 1.0% in South Africa. The proportion of total fall‐related injuries ranged from 73.3% in the Russian Federation to 44.4% in Ghana [30]. A study in India found that 38.8% of nonfatal injuries were due to falls, with one‐third occurring in people aged 60 years and over [31]. However, studies in China have reported a lower prevalence of self‐reported falls in populations [32].

In the present global study, the incidence of falls in boys initially increased and then began to decline from 2000 onwards. In girls, after a period of increase until 2003, a decreasing trend was observed until the end of the study. The mortality rate from falls has also decreased continuously in both sexes. A 2% annual decrease in the mortality rate indicates the relative success of the health system in managing and preventing the fatal consequences of falls. The results of this study are consistent with a study from 2004 to 2019 in Iran. The results showed that the crude mortality rate from unintentional injuries in men and women has had a significant decreasing trend [28]. These changes could be due to improved safety infrastructure, increased awareness of parents and adolescents, and improved health services. In Southern Sweden, there was an increasing trend in mortality from falls from 1998 to 2014 [33]. In addition, in the United States, a significant increase in annual mortality from falls was observed between 2007 and 2016 [34]. A study conducted in China found that the risk of death from unintentional falls first decreased and then increased steadily with age for both sexes, with the rate of increase accelerating in old age. The risk of death from unintentional falls was higher in Chinese men than in women across the life span [9].

Global studies also highlight significant differences between countries and age groups. Studies from Brazil, Taiwan, and China have also shown that the mortality rate from falls varies across populations, depending on age, sex, lifestyle, and environmental factors [35]. For example, in Brazil and Taiwan, the highest mortality rates are reported in the elderly and men [29], whereas in China, the annual death rate from falls is relatively lower [36]. These differences highlight the importance of interventions tailored to the cultural and social conditions of each country.

The results of the present study show that all three indicators YLL, YLD, and DALY have shown a decreasing trend during the study period. The decrease in YLL indicates a decrease in premature mortality due to falls, and the decrease in YLD also indicates a decrease in the level of disability due to falls. The decrease in DALY as a composite indicator indicates an overall improvement in the health status of adolescents in the face of falls. More than half of the total burden of disease (DALY) due to falls occurred in boys, which again emphasizes the importance of paying special attention to this group. Moreover, the contribution of YLL is higher than YLD, indicating that falls lead more to premature death than to long‐term disability; however, the contribution of YLD is also significant, and attention should be paid to rehabilitation and support for injured adolescents. This study is in line with studies conducted in Iran [37]. The risk of death and DALY due to falls varies significantly between countries, indicating that some regions of the world may not have sufficient capacity to respond to falls that lead to injury. Age is a common risk factor for falls. The prevalence, mortality, and DALYs rates from fall‐related injuries varied widely across Western European countries, with YLL rates decreasing significantly, whereas YLD rates changing a little, indicating a shift toward YLD as the main cause of DALYs from falls [38]. The difference in the results of the present study with studies from European countries is due to differences in the age subgroups studied. Older people are at higher risk for various types of injuries that can lead to death and disability, and falls are the most common cause of injury in older age groups.

In interpreting these patterns, several broader epidemiological and contextual factors should be considered. The initial increase in incidence among girls may be partly explained by changing mobility patterns, greater participation of adolescent girls in outdoor‐ and school‐based activities during the 1990s and early 2000s, and improved reporting of nonfatal injuries in this group. The substantially faster decline in mortality compared with incidence is consistent with major improvements in Iran′s health system over the past three decades, including expansion of prehospital emergency services, enhanced trauma care capacity, and increased availability of intensive care and surgical management for severe injuries. Rapid urbanization may also have influenced fall patterns by simultaneously increasing exposure to environmental hazards (e.g., construction sites and unstable urban structures) and strengthening healthcare access in urban settings. Furthermore, misclassification between RTIs and falls, particularly in earlier years when ICD coding practices were less standardized, which may account for part of the observed fluctuations in time trends. Together, these factors indicate that the observed epidemiological changes reflect not only shifts in injury occurrence but also broader demographic, environmental, and health‐system transformations in Iran [25, 26, 39].

Several factors can contribute to reducing the incidence, prevalence, and mortality of falls, including public awareness, improved safety infrastructure in schools and public places, the development of safe sports, preventive education, and better access to emergency services and treatment. However, there are still challenges such as risky behavior among adolescents, lack of parental supervision, and weaknesses in some urban and rural infrastructure that need to be addressed. The findings of this study are consistent with global studies, such that in most countries, unintentional falls in adolescents are decreasing, but they remain one of the leading causes of death and disability in this age group [40]. Gender differences, the role of environmental and social factors, and the importance of education and prevention are emphasized in all studies.

The strengths of this study include the use of globally standardized GBD data and the analysis of long‐term temporal trends, which allow international comparisons and assessment of policy effectiveness. However, several important limitations must be acknowledged to ensure transparent interpretation of the results. First, GBD modeled estimates depend heavily on the quality, completeness, and diagnostic accuracy of the underlying data sources. Because the GBD integrates heterogeneous datasets from different years and regions, issues such as underreporting and incomplete injury documentation may influence the accuracy of the estimates. Second, misclassification of ICD codes, particularly within the W00–W19 range used to define falls, is possible. Falls may be miscoded as other types of injuries, or vice versa, especially in earlier years when coding systems and reporting practices were less standardized. Third, the ecological design of this study limits causal inference. Although population‐level estimates are suitable for identifying temporal patterns and broad associations, they cannot determine causality at the individual level and may obscure important within‐country heterogeneity. Fourth, the lack of subnational data in the GBD database prevents analysis of regional variations within Iran. Differences between provinces, rural and urban settings, or local socioeconomic contexts remain unexamined due to the absence of geographically granular data. Finally, the GBD dataset does not provide detailed information on injury mechanisms, such as the specific type of fall (e.g., fall from height, slip, and trip), the precise location where the event occurred (home, school, and public space), or contextual socioeconomic factors. This lack of detail limits the ability to conduct more nuanced subgroup analyses and identify high‐risk contexts. These limitations should be considered when interpreting the findings.

## 5. Conclusion

Unintentional falls remain one of the most important causes of death and disability in Iranian adolescents, although decreasing trends in their incidence, prevalence, and mortality have been observed over the past three decades. Special attention to high‐risk groups, especially adolescent boys, strengthening safety education, improving infrastructure, and expanding health and rehabilitation services are essential. Continued epidemiological surveillance and prospective research to identify emerging risk factors and evaluate the effectiveness of interventions are key to maintaining and accelerating the current downward trend. Policymakers should draw on successful domestic and international experiences to design prevention and care programs that further reduce the burden of disease caused by falls in adolescents.

## Funding

No funding was received for this manuscript.

## Ethics Statement

This observational study was approved by the Ethics Committee of Jahrom University of Medical Sciences (IR.JUMS. REC.1404.039), who we acknowledge.

## Consent

We have utilized the public “Global Burden of Disease Study” (GBD) database according to the proposed format of the “Institute for Health Metrics and Evaluation” (IHME). In addition, the results are published as a group, and there is no access to individual information.

## Conflicts of Interest

The authors declare no conflicts of interest.

## Data Availability

The data that support the findings of this study are openly available in INSTITUTE FOR HEALTH METRICS AND EVALUATION at https://ghdx.healthdata.org/gbd-results-tool.
